# Negative calcium balance despite normal plasma ionized calcium concentrations during citrate anticoagulated continuous venovenous hemofiltration (CVVH) in ICU patients

**DOI:** 10.1007/s40620-022-01482-y

**Published:** 2022-11-07

**Authors:** Evert de Jonge, Marije van der Vooren, Judith M. E. P. Gillis, Michael R. del Prado, Jeanette Wigbers, Ferishta Bakhshi-Raiez, Carlos V. Elzo Kraemer

**Affiliations:** 1 Department of Intensive Care, Leiden University Medical Center, Albinusdreef 2, 2333 ZA Leiden, The Netherlands; 2 Department of Clinical Chemistry, Leiden University Medical Center, Leiden, The Netherlands; 3 Department of Clinical Informatics, Amsterdam University Medical Center, Amsterdam, The Netherlands

**Keywords:** Renal replacement therapy, CVVH, Citrate, Calcium, Fractures

## Abstract

**Background:**

Supplementation of calcium during continuous venovenous hemofiltration (CVVH) with citrate anticoagulation is usually titrated using a target blood ionized calcium concentration. Plasma calcium concentrations may be normal despite substantial calcium loss, by mobilization of calcium from the skeleton. Aim of our study is to develop an equation to calculate CVVH calcium and to retrospectively calculate CVVH calcium balance in a cohort of ICU-patients.

**Methods:**

This is a single-center retrospective observational cohort study. In a subcohort of patients, all calcium excretion measurements in patients treated with citrate CVVH were randomly divided into a development set (*n* = 324 in 42 patients) and a validation set (*n* = 441 in 42 different patients). Using mixed linear models, we developed an equation to calculate calcium excretion from routinely available parameters. We retrospectively calculated calcium balance in 788 patients treated with citrate CVVH between 2014 and 2021.

**Results:**

Calcium excretion (mmol/24 h) was − 1.2877 + 0.646*[Ca]_blood,total_ * ultrafiltrate (l/24 h) + 0.107*blood flow (ml/h). The mean error of the estimation was − 1.0 ± 6.7 mmol/24 h, the mean absolute error was 4.8 ± 4.8 mmol/24 h. Calculated calcium excretion was 105.8 ± 19.3 mmol/24 h. Mean daily CVVH calcium balance was − 12.0 ± 20.0 mmol/24 h. Mean cumulative calcium balance ranged from − 3687 to 448 mmol.

**Conclusion:**

During citrate CVVH, calcium balance was negative in most patients, despite supplementation of calcium based on plasma ionized calcium levels. This may contribute to demineralization of the skeleton. We propose that calcium supplementation should be based on both plasma ionized calcium and a simple calculation of calcium excretion by CVVH.

**Graphical abstract:**

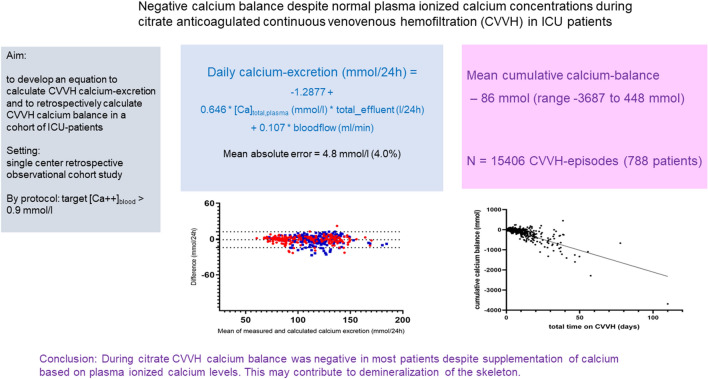

**Supplementary Information:**

The online version contains supplementary material available at 10.1007/s40620-022-01482-y.

## Introduction

Systemic anticoagulation during continuous venovenous hemofiltration (CVVH) is associated with bleeding complications. Citrate-based regional anticoagulation has a lower risk of bleeding [1], is associated with prolonged filter survival time [2] and is increasingly used in ICUs all over the world. Citrate is administered pre-filter leading to chelation of calcium with low plasma concentrations of ionized calcium and inhibition of coagulation in the extracorporeal circuit. Excessively low levels of blood ionized calcium are avoided by administering calcium in the substitution fluid or as intravenous solution. Most often, the rate of calcium supplementation is guided by the blood concentration of ionized calcium. In present guidelines, aiming for ionized calcium levels higher than 1.0 mmol/l, much lower than normal values, is advocated [3, 4], but there is no proof that these target levels are appropriate. Even lower target levels of plasma ionized calcium, accepting values as low as 0.8–0.9 mmol/l, were used in recent studies [5, 6]. Unfortunately, maintaining plasma ionized calcium concentrations within targeted values does not rule out the risk of net calcium loss from the body. Even with a negative net calcium balance, homeostatic mechanisms may keep ionized calcium concentrations within normal limits by mobilizing calcium from the skeleton. Demineralization of the skeleton, however, may lead to osteopenia and should be avoided.

An alternative for guiding calcium supplementation by blood ionized calcium concentrations is monitoring the daily loss of calcium by CVVH. This is possible by measuring the calcium concentration in the ultrafiltrate. Alternatively, daily calcium excretion in the ultrafiltrate could be calculated using routinely available CVVH parameters and the blood calcium concentration.

In the published literature, we were able to find two mathematical models to calculate calcium loss during citrate CVVH, both from the same study group in Shanghai, China. Neither of these models have been validated in independent populations of patients [7, 8].

Aim of this study is to develop and to validate an equation to calculate calcium loss in CVVH ultrafiltrate using routinely available CVVH parameters and blood calcium concentration, and to apply this equation to calculate the effects of CVVH on calcium losses and calcium balance in a retrospective cohort of ICU patients treated with CVVH with citrate anticoagulation.

## Methods

This study consisted of two parts. First a model was developed to calculate calcium excretion by CVVH and, second, this model was used to retrospectively study calcium balance in CVVH patients during a 7-years period. The development and validation of a model to calculate calcium excretion by CVVH was performed as a retrospective cohort study in all consecutive patients, 18 years or older, treated with CVVH and citrate anticoagulation in the ICU of the Leiden University Medical Center (LUMC) from January 1st, 2021 until December 31st, 2021. The LUMC has a 24-bed mixed medical and surgical ICU.

The retrospective calculation of calcium losses and calcium balance in ICU patients on citrate anticoagulated CVVH was performed in all consecutive patients, 18 years or older, treated with CVVH and citrate anticoagulation in the ICU of the Leiden University Medical Center (LUMC) from January 1st, 2014 until July 1st, 2021.

As part of our local CVVH protocol, during 2021, total calcium was measured in an ultrafiltrate sample taken at 6:00 a.m. (EST tube, prod. no: 362725, Becton Dickinson, Rutherford, N.J., USA) on a Cobas 8000 Modular c702 system (Roche Diagnostics; Mannheim, Germany). For this study, we used measurements of total calcium and free calcium determined in the blood at the same time. Total calcium was analyzed in serum (3.5 mL SST(TM) II PET tube with clot activator (silica) and separating gel, prod. no: 367957, Becton Dickinson) using the Cobas 8000 Modular c702 system. Ionized calcium was analyzed in heparin-balanced blood (blood gas syringes RAPIDLyte, prod. no: 00925045, Siemens Healthcare, Sudbury, UK) by an ion-selective electrode method using the RAPIDPoint 500 Blood Gas System (Siemens Healthcare, Erlangen, Germany). Before analysis on the Cobas 8000 systems, samples were centrifuged (RCF 3000*g*, 8 min., 22 °C). CVVH parameters such as substitution flow, blood flow, fluid withdrawal by CVVH, calcium supplementation, and citrate dose were registered every hour in the Patient Data Management System.

Citrate CVVH was performed using Prismaflex^®^ or Prismax^®^ equipment and either the HF1400 hemofilter (polyarylethersulfone (PAES) membrane, 1.4 m^2^) or ST150 hemofilter (AN 69 ST membrane, 1.5 m^2^) (Baxter international inc. Deerfield IL, USA). Citrate was administered pre-filter as Prismocitrate 18/0^®^, containing 18 mmol of citrate per liter as well as sodium (140 meq/L) and chloride (86 meq/L). At initiation of CVVH, citrate was started at a rate of 2.4 mmol/liter blood concentration in the circuit (8 ml prismocitrate 18/0 per ml/h blood flow). The rate of citrate administration could be lowered according to our local protocol if the ratio of free calcium to total calcium decreased, and citrate dose could be increased in case of frequent clotting of the circuit. As substitution fluids we used Biphozyl^®^ (Baxter Holding B.V, Utrecht, the Netherlands) containing no calcium, Phoxilium^®^ (Baxter Holding B.V, Utrecht, the Netherlands) containing 1.25 mmol/l calcium or Prismasol^®^ (Baxter Holding B.V, Utrecht, the Netherlands) containing 1.75 mmol calcium per liter. Additionally all patients also received calcium supplementation given as magnesium (24 mmol/l)-calcium (54 mmol/l) chloride solution intravenously, titrated on a target ionized calcium concentration between 0.9 and 1.1 mmol/l (from January 1st, 2014 until December 31st 2020) or between 1.15 and 1.30 mmol/l (since January 1st, 2021). Free calcium was measured four times daily.

According to our protocols, patients received Nutrison Protein Plus enteral feeding (Nutricia, Zoetermeer, the Netherlands), 1 ml/kg body weight/h, containing 22 mmol calcium per liter) or 1 ml/kg/h parenteral feeding (SmofKabiven, Fresenius Kabi, Huis ter Heide, the Netherlands) containing 2.53 mmol calcium per liter.

### Statistical analysis

Statistical analyses were made in SPSS version 25.0. For the development of the equation to calculate calcium loss by CVVH ultrafiltrate, the population of patients treated with CVVH in 2021 was randomly divided into a development and a validation set, both containing all measurements of a subset of 50% of patients. A mixed linear model was constructed with CVVH calcium excretion as a dependent variable, ultrafiltrate volume times total calcium concentration in plasma (UF*[Ca]_total,plasma_), blood flow, dose of citrate, weight, ratio of pre-dilution to post-dilution and used filter (HF1400 or ST150) as fixed effect variables, and patient as a random effect variable. Fixed effect variables were kept in the model if they added to the performance with a *p* value < 0.10.

The performance of the built model was validated in the independent validation set and reported as the mean of the absolute errors (differences between measured and predicted values). Differences between means in subgroups by blood flow, pre-dilution/post-dilution ratio and plasma ionized calcium were analyzed by one-way Anova. In the same validation population, we validated two models that were previously published. One model by Yu and coauthors: Calcium excretion by CVVH (mmol/h) = (0.0006938*total_ultrafiltrate(ml/min) + 0.7983)*calcium(mmol/l)*(60/1000)*(21.42 + 0.35*total_ultrafiltrate(ml/min)) [7], and one model by Zheng and coauthors: Calcium excretion by CVVH (mmol/h) = ((0.0006938*total_ultrafiltrate(ml/min) + 0.7983) * blood flow (L/h)) * (1 − Hematocrit) * ultrafiltrate (L/h))/(blood flow (l/h) + pre-dilution fluid (l/h)) [8].

For the retrospective analysis of calcium balance during CVVH in ICU patients admitted from 2014 until July 2021, CVVH settings were retrieved on an hourly basis from the Patient Data Management System (Metavision, iMD soft, Tel Aviv, Israel). For calculation of the calcium excretion in the CVVH ultrafiltrate, we applied the equation that we developed and validated in the first part of this study. Administered calcium was calculated as the sum of calcium in the substitution fluid and calcium given intravenously. CVVH calcium balance was calculated as administered calcium minus the calcium excretion in ultrafiltrate without taking into consideration the amount of calcium lost in urine and stools and calcium intake via feeding.

## Results

From January 1st, 2021 until December 31st, 2021, 765 daily measurements of calcium excretion in CVVH ultrafiltrate in 84 patients were analyzed for this study. Characteristics of patients and the CVVH episodes are shown in Table 1.

**Table 1:** Characteristics of analyzed CVVH episodes (days) and patients used for developing the model to calculate calcium loss by CVVH ultrafiltrate (development) and for validation of the model

Variable	Development	Validation
No of measurements	324	441
No of patients	42	42
Male (%)	72	73
Age (mean ± SD)	60 ± 10	60 ± 10
Weight (mean ± SD)	94 ± 24	81 ± 18
CVVH-filter		
HF1400	162	213
ST150	162	228
Blood flow CVVH (ml/min)	202 ± 285	186 ± 34
Substitution fluid		
Biphozyl	214	275
Phoxilium	63	118
Prismasol	47	47
Ultrafiltrate (L/h)	2.6 ± 0.4	2.4 ± 0.4
Citrate dose (mmol/l.blood)	2.56 ± 0.24	2.60 ± 0.23
Blood [Ca]_total_ (mmol/l, mean ± SD)	2.44 ± 0.20	2.42 ± 0.25
Blood [Ca^++^] (mmol/l, mean ± SD)	1.20 ± 0.09	1.20 ± 0.11
Calcium-excretion ultrafiltrate (mmol/24 h, mean ± SD)	120 ± 21	111 ± 23
Calcium supplementation (mmol/24 h,intravenous and in substitution fluid)	125 ± 25	113 ± 32.6

In the development set, using a mixed linear model, we found a linear association between daily calcium excretion in the ultrafiltrate (dependent variable) and the product of plasma calcium and ultrafiltration flow ([Ca]_blood, total_ * UF) and CVVH blood flow with the following equation: Daily calcium-excretion (mmol/24 h) = − 1.2877 + 0.646 * [Ca]_total,plasma_ (mmol/l) * (total_effluent (l/24 h)) + 0.107 * blood flow (ml/min).

Adding the potential covariates, i.e., citrate dose, ratio of pre-dilution to post-dilution, and used filter (HF1400 or ST150), did not improve the fit of the model (Supplementary Table 1).

**Fig. 1:**
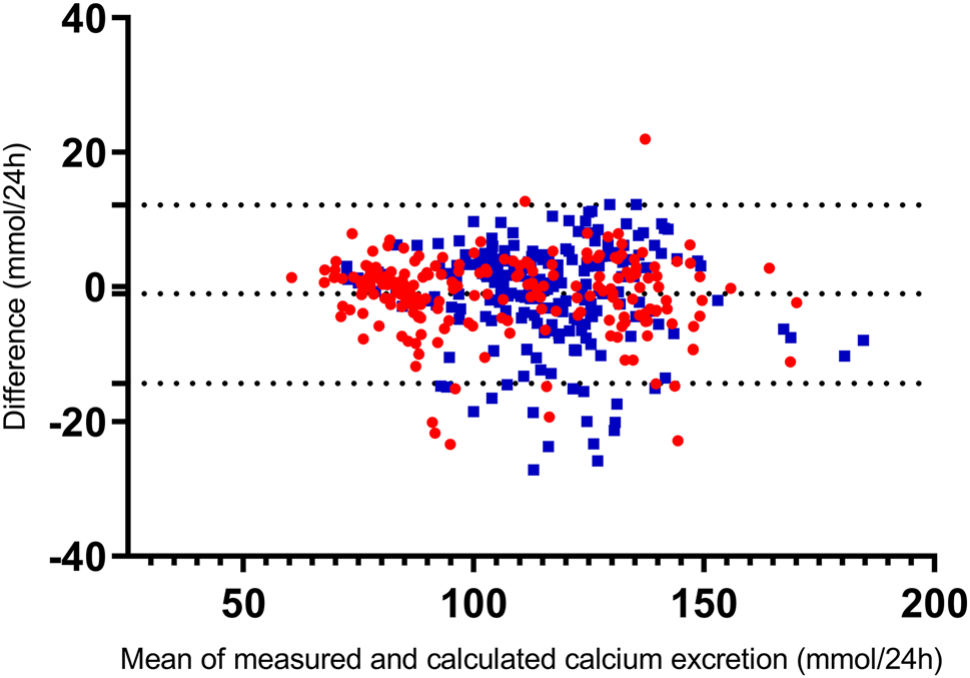
Bland–Altman plot showing the association between the difference between calculated calcium excretion and measured calcium excretion in ultrafiltrate (*y*-axis) and the mean of calculated and measured calcium excretion (*x*-axis). Horizontal lines represent mean and 95% confidence interval. Calculated calcium excretion by CVVH is 1.2877 + 0.646 * [Ca]_total, blood_ (mmol/l) * total_effluent (l/24 h) + 0.107 * blood flow (ml/h)) in the validation population. Blue circles indicate measurements with the ST150 filter (*n* = 228). Red circles represent measurements with the HF1400 filter (*n* = 213)

Figure 1 and supplemental Table 2 show the performance in the validation set for our model as well as for the two previously published models. The mean calculated calcium excretion minus the measured calcium excretion (error) was − 1.05 ± 6.7 (*p* = 0.01) in our model, − 8.53 ± 13.4 (*p* < 0.001) in the model by Yu and coauthors and − 12.8 ± 7.3 (*p* < 0.001) in the model by Zheng and coauthors. The mean of the absolute error was 4.8 ± 4.8, 13.0 ± 9.1 and 12.9 ± 7.2 for the new model, the model by Yu and the model by Zheng, respectively. The absolute error of our newly developed model in subgroups by used filter, plasma ionized calcium, CVVH blood flow, percentage pre-dilution and citrate dose is given in Supplementary table 3.

From January 1st, 2014 until July 1st, 2021, 788 patients were treated with CVVH and citrate anticoagulation (Table 2). Hospital survival was 52%. Mean duration of CVVH was 6.3 ± 7.8 days in patients who survived up to hospital discharge and 7.1 ± 11.0 days in non-survivors.

**Table 2:** Characteristics of patients (*n* = 788) in a retrospective study on calcium losses by CVVH-ultrafiltrate and on CVVH calcium balance

Variable	Survivors	Non-survivors
Number of patients	406	382
Female (%)	126 (31)	145 (38)
Weight ± SD (kg)	82 ± 20	81 ± 19
Age ± SD (y)	60 ± 13	63 ± 13
Comorbidities (%)		
Circulatory insufficiency	15 (3.7)	20 (5.2)
Respiratory insufficiency	8 (2.0)	9 (2.4)
Cirrhosis	24 (5.9)	32 (8.4)
Metastasized cancer	5 (1.2)	6 (1.6)
Hematological malignancy	14 (3.4)	48 (12.6)
Chronic renal failure	105 (25.9)	69 (18.1)
Chronic dialysis	37 (9.1)	21 (5.5)
Referring specialty (%)		
Internal medicine	83 (20.4)	60 (15.8)
Surgery	88 (21.7)	72 (18.8)
Pulmonology	14 (3.4)	13 (3.4)
Cardiology	41 (10.1)	43 (11.3)
Thoracic surgery	120 (29.6)	82 (21.5)
Neurology	5 (1.2)	4 (1.0)
APACHE IV score (± SD)	88 ± 26	102 ± 33
APACHE IV predicted mortality	0.34 ± 0.25	0.48 ± 0.29
ICU length-of-stay (days ± SD)	19.7 ± 19.6	17.7 ± 20.2
Days on CVVH (± SD)	6.3 ± 7.8	7.1 ± 11.0

During the study period, 129,752 CVVH episodes (days) were analyzed, 127,897 with the HF1400 filter and 1855 with the ST150 filter. Substitution fluid was Biphozyl (*n* = 5124), Phoxilium (*n* = 115,263), Prismasol 2 (*n* = 9281) and Prismasol 4 (*n* = 84). Mean (SD) blood flow was 187 ± 31 ml/h. Citrate was administered at a dose of 2.25 ± 0.18 mmol/liter blood. Ultrafiltrate flow was 2599 ± 438 ml/h. [Ca]_total,blood_ was 2.16 ± 0.20 mmol/l. [Ca^++^]_blood_ was 1.06 ± 0.08 mmol/l.

Calculated calcium excretion by CVVH was 105.8 ± 19.3 mmol/24 h. Calcium administration by substitution fluid and by intravenous infusion was 92.9 ± 23.9 mmol/24 h. The CVVH calcium balance was − 12.0 ± 20.0 mmol/24 h.

**Fig. 2:**
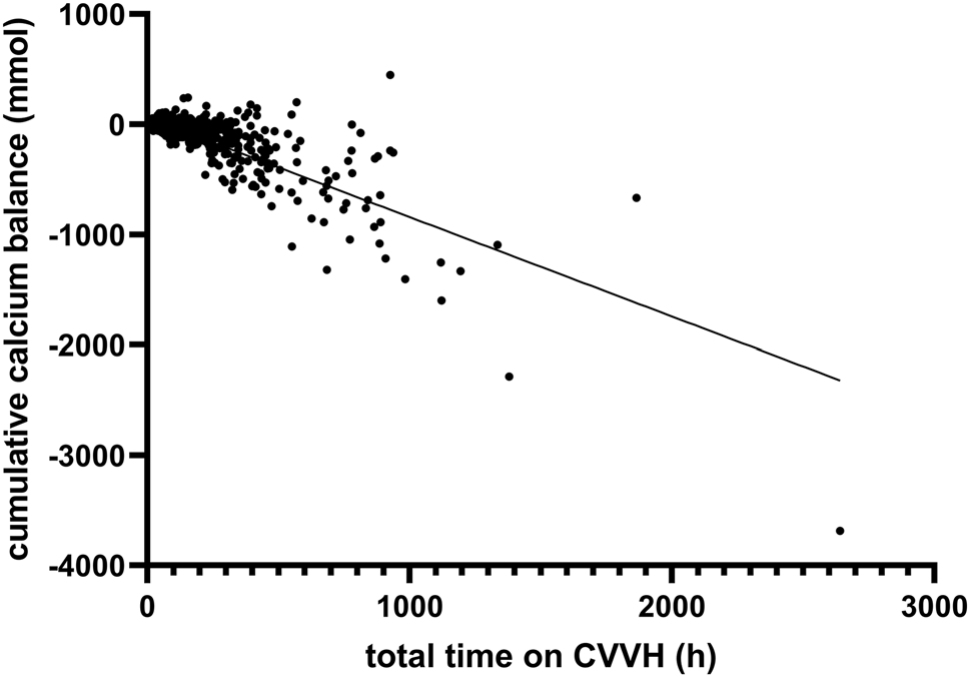
Cumulative CVVH calcium-balance by time on CVVH (hours) in 788 patients treated with CVVH in the ICU. Calcium-balance = calcium supplementation by CVVH substitution fluid + intravenous calcium administration – calcium loss in ultrafiltrate. Calcium loss (mmol/24 h) calculated as − 1.279 + 0.646 * [Ca]_blood,total_ (mmol/l) * total_effluent (l/24 h) + 0.107 * bloodflow (ml/h)

The mean number of CVVH days per patient was 6.7 ± 9.4 days. Cumulative CVVH calcium balance was − 86.3 ± 251.4 mmol (range − 3687 to 448 mmol). Cumulative CVVH calcium balance by time on CVVH is shown in Fig. 2.

**Fig. 3:**
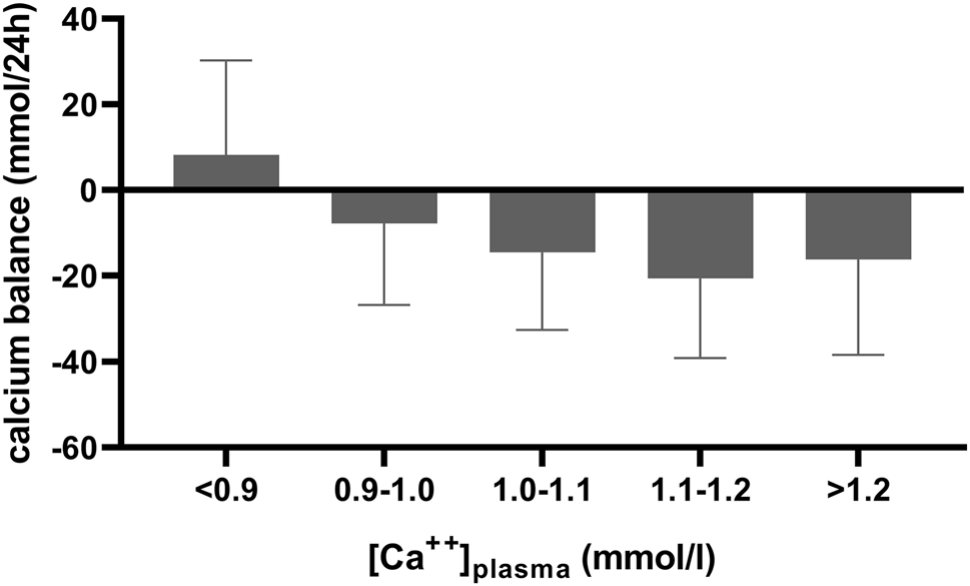
Mean and SD of daily CVVH calcium balance by plasma concentration of ionized calcium in patients admitted to the ICU from January 1st, 2014 until December 31st, 2020 with target [Ca^++^] > 0.9 mmol/l. Calcium-balance = calcium supplementation by CVVH

In a population of patients admitted from 2014 to 2020, all treated using a protocol with calcium supplementation aiming at a target plasma ionized calcium greater than 0.9 mmol/l, the calculated daily mean calcium excretion by CVVH was higher with increasing plasma ionized calcium concentrations (data not shown). The mean daily CVVH calcium balance in subgroups by blood ionized calcium concentrations is shown in Fig. 3.

In patients with a [Ca^++^]_blood_ lower than the target concentration of 0.9 mmol/l a positive CVVH calcium balance was present. In patients with a [Ca^++^]_blood_ higher than 0.9 mmol/l, CVVH calcium balance was more negative with higher blood ionized calcium concentrations.

The mean [Ca]_total,blood,_ [Ca^++^]_blood_, Calcium excretion in ultrafiltrate and Calcium supplementation per year (2014–2021) is shown in supplementary table 2 and supplementary Fig. 1. From January 1st, 2021 the protocol for calcium supplementation was changed aiming at a higher [Ca^++^]_blood_ 1.15 – 1.3 mmol/l. In 2021, compared to the period between 2014 and 2020, calcium losses in ultrafiltrate were similar, whereas plasma calcium concentrations, and calcium supplementation were higher. Notably, from 2014 to 2020, CVVH calcium balance ranged from − 17.1 ± 20.6 to − 10.0 ± 18.3 mmol/24 h, in 2021 it was 8.1 ± 22.2 mmol/24 h in 2021.

## Discussion

In this study we present a simple mathematical equation to calculate calcium-excretion in the ultrafiltrate during CVVH with citrate anticoagulation using routinely available parameters. Using this equation, we found that in a population of ICU patients treated with citrate anticoagulated CVVH, cumulative CVVH calcium balance was negative in the majority of patients even if plasma concentrations of ionized calcium were normal and well above the commonly advocated target level of 1.0 mmol/l [5, 6].

Our mathematical model fits the data very well. In an independent, separate population from our ICU, the mean absolute error in the estimation of calcium-loss by CVVH was 4.8 mmol/24 h (4.5% of mean daily calcium excretion). We found that the performance of our model was better in patients treated with the HF1400 filter than in patients treated with the ST150 filter, but the difference was only small (mean absolute error 4.0 ± 4.2 mmol/24 h vs. 5.6 ± 5.2 mmol/24 h, *p* < 0.01). Both filters used in our study had similar surface area (1.4 and 1.5 m^2^). Performance may differ more when using filters that have larger differences in surface area. The performance of the model was similar in subgroups by blood flow, dose of citrate, percentage of substitution by pre-dilution and blood calcium concentration.

The performance of our model was better than that of two different mathematical models that were previously published [7, 8]. The difference may be explained by the population in which the models were validated. Although the performance of our new model was determined in different patients than where the model was developed, it was still assessed in patients from the same ICU who were treated using the same equipment and protocols as in the validation set. In contrast, the model from the literature was developed in a different patient population from another country with a slightly different treatment protocol, using an AN69 filter with an area of 0.9 m^2^, smaller than the 1.4–1.5 m^2^ used in our ICU. The performance of the previously published model has not been validated before, so we do not know the performance of that model in the setting where it was developed.

We found that in ICU patients, CVVH with citrate anticoagulation and calcium supplementation guided by the plasma ionized calcium concentration resulted in a negative CVVH calcium balance of mean − 12.5 ± 20.4 mmol/24 h. The mean cumulative CVVH calcium balance was dependent of the time on CVVH. Five percent of patients had a cumulative CVVH calcium balance that was more negative than − 475 mmol (19 g). Normal total body calcium is approximately 1000*g* in normal subjects [9] and is 82% of normal in women with osteoporosis [10]. The average yearly loss of total body calcium is approximately 3.7 g in pre-menopausal women and 11 g in post-menopausal women [11]. Thus, the added loss of calcium by citrate-CVVH may be substantial with respect to the risk of osteoporosis. Critical illness is associated with an increase in bone resorption, and loss in bone mineral density continues during the first two years after intensive care [12–14]. Calcium loss by CVVH will add to the risk of osteopenia and fragility fractures.

During the major part of this study we adhered to a target ionized calcium concentration of 0.9–1.1 mmol/l, in accordance with recommendations in the literature [3, 4]. Interestingly, calcium balance was most negative in patients with the highest plasma ionized calcium concentrations. This is in accordance with the linear association between calcium excretion and plasma calcium as presented in our mathematical equation. Plasma calcium concentrations may remain normal despite calcium losses due to the body’s homeostatic mechanisms, most importantly the production of parathormone (PTH). So, loss of calcium-citrate complexes in the CVVH ultrafiltrate will lead to increased PTH secretion, thus normalizing plasma ionized calcium concentration at the cost of calcium loss from the skeleton [5]. Also, patients may have increased plasma calcium concentrations due to immobility. If so, calcium excretion by CVVH will be increased while little calcium will be supplemented because plasma calcium concentrations remain higher than the target concentrations used for guiding calcium administration.

During the last 6 months of our study, due to a change in our local CVVH protocol, we adhered to a higher ionized calcium target of 1.15–1.30 mmol/l. Interestingly, mean plasma concentrations of ionized calcium were only slightly higher compared with the period with lower target plasma calcium, but the rate of calcium supplementation was higher and CVVH calcium balance changed from negative to positive. The likely explanation for this is that plasma calcium levels were hardly determined by the rate of calcium supplementation but much more by homeostatic mechanisms of the body [15, 16]. Consequently, targeting a plasma calcium level of 1.15–1.3 mmol/l, slightly higher than the body’s own homeostatic setpoint, led to higher calcium intake without increasing the plasma calcium concentration and thus without increasing calcium losses by CVVH. The important message is that a normal or even high plasma ionized calcium concentration should not reassure us regarding potential loss of calcium during citrate CVVH. Indeed, a case report has been published of a patient on continuous renal replacement therapy with citrate anticoagulation who managed to have normal blood ionized calcium concentrations without any exogenous calcium supplementation but with severe osteopenia and several bone fractures on radiologic examinations [17]. As discussed above, calcium supplementation should be guided by parameters other than normal plasma ionized calcium concentration alone. A mathematical model may provide an estimation of calcium loss using only parameters that are already routinely available. If integrated in patient data management systems, it may easily present the actual CVVH calcium balance. Calcium administration could then be titrated on both calcium balance to prevent calcium loss from the skeleton and plasma ionized calcium concentration to avoid hypo/ or hypercalcemia. Future studies should focus on the effects of monitoring calcium balance on clinically relevant endpoints. Osteoporosis may be an underestimated adverse outcome after treatment in the intensive care unit with citrate CVVH that could be avoided by aiming at a positive calcium balance. On the other hand, however, we can not rule out the possibility that too much calcium supplementation can have negative effects on inflammatory processes [18] and specifically on calcium precipitation in the vessel walls and acceleration of atherosclerosis.

Some limitations of our study should be discussed. First, from a theoretical point of view, it is likely that factors not included in our model could influence calcium excretion during CVVH. The concentration of citrate used for anticoagulation will influence the ratio of protein-bound and non protein-bound calcium in the blood of the CVVH circuit and thereby the filtration of calcium to the ultrafiltrate. Likewise, the sieving coefficient of a filter is influenced by the amount of time a filter is in use. However, citrate dose and type of filter did not add to the performance of our model, and the results of our validation show that the influence of these factors is very limited. Second, validation was performed in a separate patient population, but treated in the same ICU and using the same equipment and protocols. Before using the model in other settings, its performance should be confirmed in those circumstances. This could be easily done by direct measurement of total calcium concentration in the ultrafiltrate in a number of patients. Finally, in our calculation of calcium balance we only considered calcium losses by CVVH and calcium intake if given intravenously or as part of the CVVH substitution fluids. There is additional intake by enteral or parenteral feeding and some extra losses in the urine if there is some residual kidney function. However, in a study in trauma patients in the ICU calcium losses in urine were low (mean 0.2 mmol/day in patients on renal replacement therapy and mean 3.2 mmol/day in patients without renal replacement therapy) and negligible compared to losses in the CVVH ultrafiltrate [19].

## Conclusion

During citrate anticoagulated CVVH, and following recommendations for calcium supplementation from the literature, a negative calcium balance was commonly found. Adhering to higher target levels for plasma ionized calcium will lower this risk, but can not prevent negative calcium balances in patients who have some degree of hypercalcemia, as is frequently present in ICU patients. We present a validated formula to easily calculate calcium excretion in the CVVH ultrafiltrate from plasma calcium concentrations, ultrafiltrate flow and CVVH blood flow. Our equation may be used at the bedside to help avoid calcium loss from the skeleton.

## Supplementary Information

Below is the link to the electronic supplementary material.

Supplementary figure 1. Mean daily CVVH calcium balance by year of ICU admission. From 2014 to 2020 the target plasma ionized calcium was 0.9–1.1 mmol/l. From January 1, 2021, it was changed to 1.15 to 1.3 mmol/l. Error bars represent SD. *P*<0.001 for differences between 2014–2020 and 2021 by unpaired *t*-test (TIF 979 kb)

Supplementary file2 (DOCX 14 kb)

Supplementary file3 (DOCX 12 kb)

Supplementary file4 (DOCX 14 kb)

## Data Availability

The datasets analyzed during the current study are available from the corresponding author on reasonable request.
