# Supplementary figures and images for: Negative calcium balance despite normal plasma ionized calcium concentrations during citrate anticoagulated continuous venovenous hemofiltration (CVVH) in ICU patients
Source: J Nephrol. 2022 Nov 7;36(4):1019–26. doi: 10.1007/s40620-022-01482-y (PMC10227114; doi:10.1007/s40620-022-01482-y)

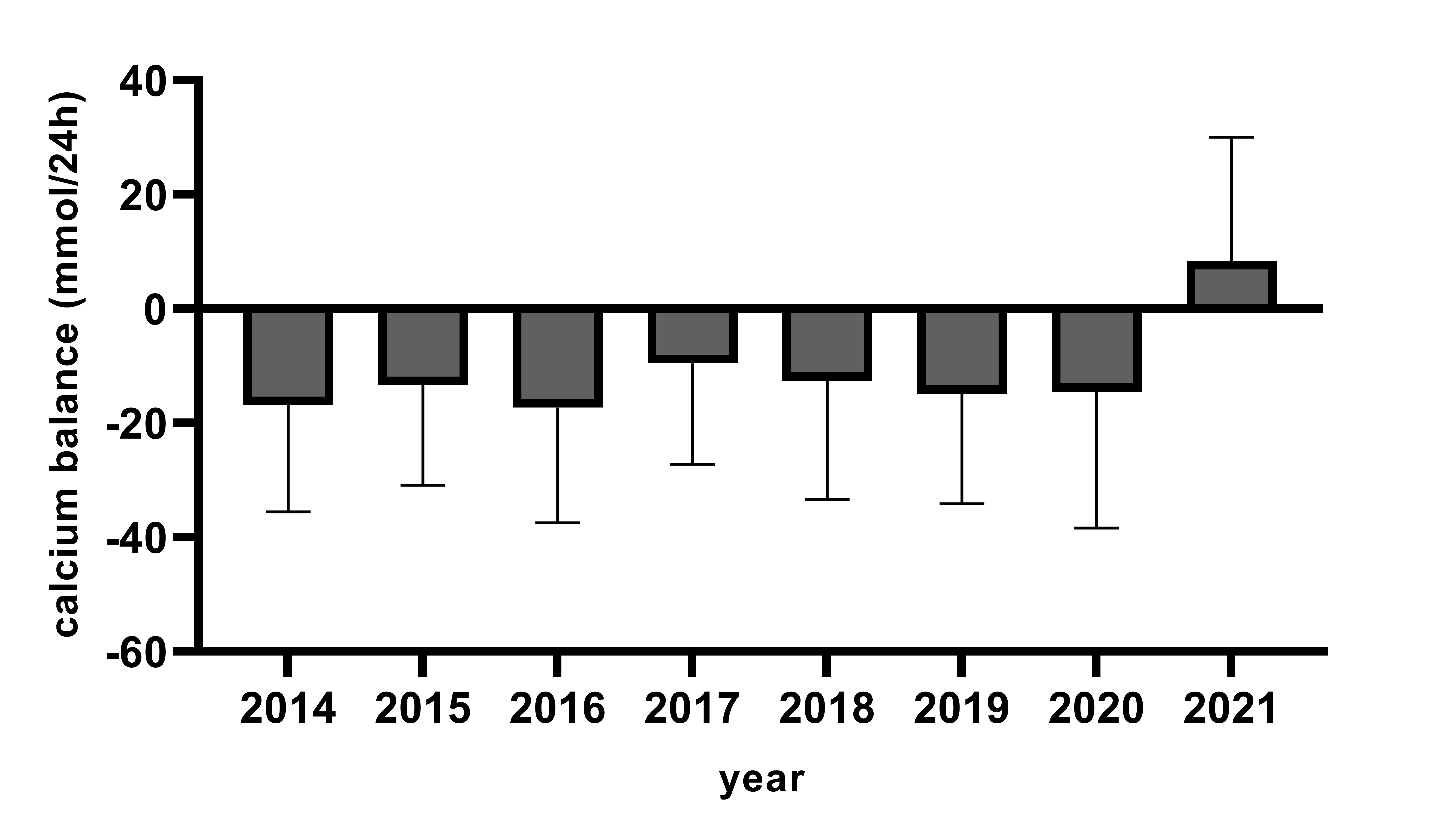

Supplement: Supplementary file 1 — Supplementary figure 1. Mean daily CVVH calcium balance by year of ICU admission. From 2014 to 2020 the target plasma ionized calcium was 0.9–1.1 mmol/l. From January 1, 2021, it was changed to 1.15 to 1.3 mmol/l. Error bars represent SD. P<0.001 for differences between 2014–2020 and 2021 by unpaired t-test (TIF 979 kb) [file 40620_2022_1482_MOESM1_ESM.tif]
