# Supplementary material for: Negative calcium balance despite normal plasma ionized calcium concentrations during citrate anticoagulated continuous venovenous hemofiltration (CVVH) in ICU patients
Source: J Nephrol. 2022 Nov 7;36(4):1019–26. doi: 10.1007/s40620-022-01482-y (PMC10227114; doi:10.1007/s40620-022-01482-y)
Supplement: Supplementary file 2 — Supplementary file2 (DOCX 14 kb) [file 40620_2022_1482_MOESM2_ESM.docx]

|  | | | | | | | |
| --- | --- | --- | --- | --- | --- | --- | --- |
| Parameter | Estimate | Std. Error | df | t | Sig. | 95% Confidence Interval | |
|  |  |  |  |  |  | Lower Bound | Upper Bound |
| Intercept | 3,762805 | 7,919313 | 137,574 | ,475 | ,635 | -11,896511 | 19,422120 |
| [Ca]_blood, total_ * UF | ,639292 | ,027707 | 303,257 | 23,073 | ,000 | ,584769 | ,693816 |
| Blood flow | ,139230 | ,043848 | 241,064 | 3,175 | ,002 | ,052856 | ,225605 |
| Citrate dose | 2,912829 | 4,776935 | 306,858 | ,610 | ,542 | -6,486865 | 12,312524 |
| Body weight | -,067979 | ,059868 | 50,304 | -1,135 | ,262 | -,188208 | ,052251 |
| Percentage _pre-dilution | -16,500873 | 19,602856 | 297,396 | -,842 | ,401 | -55,078760 | 22,077015 |
| Filter HF1400 | -1,728905 | 1,659887 | 85,751 | -1,042 | ,301 | -5,028788 | 1,570979 |
| a. Dependent Variable: measured Calcium in ultrafiltrate. | | | | | | | |

Supplementary table 1. Estimates of fixed effects of [Ca]_blood, total_ * UF, blood flow, citrate dose, body weight, percentage pre-dilution and use of HF1400 filter on measured calcium excretion in ultrafiltrate by Linear Mixed Model.
