# Supplementary material for: Negative calcium balance despite normal plasma ionized calcium concentrations during citrate anticoagulated continuous venovenous hemofiltration (CVVH) in ICU patients
Source: J Nephrol. 2022 Nov 7;36(4):1019–26. doi: 10.1007/s40620-022-01482-y (PMC10227114; doi:10.1007/s40620-022-01482-y)
Supplement: Supplementary file 3 — Supplementary file3 (DOCX 12 kb) [file 40620_2022_1482_MOESM3_ESM.docx]

|  | Present model | Model by Yu [8] | Model by Zheng [9] |
| --- | --- | --- | --- |
| Calcium loss estimation by model (mmol/24h; mean ± SD) | 110.2 ± 22.4 | 102.8 ± 13.6 | 98.4 ± 21.4 |
| Measured calcium loss (mmol/24h; mean ± SD) | 111.8 ± 24.1 | 111.8 ± 24.1 | 111.8 ± 24.1 |
| Error* (mean ± SD) | -1.05 ± 6.7 | -8.5 ± 13.4 | -12.8 ± 7.3 |
| Absolute error (mmol/24h, mean ± SD) | 4.8 ± 4.8 | 13.0 ± 9.1 | 12.9 ± 7.2 |

*Supplementary Table 2. Performance in the validation set for the newly developed model and two previously published models..*
