# Supplementary material for: Negative calcium balance despite normal plasma ionized calcium concentrations during citrate anticoagulated continuous venovenous hemofiltration (CVVH) in ICU patients
Source: J Nephrol. 2022 Nov 7;36(4):1019–26. doi: 10.1007/s40620-022-01482-y (PMC10227114; doi:10.1007/s40620-022-01482-y)
Supplement: Supplementary file 4 — Supplementary file4 (DOCX 14 kb) [file 40620_2022_1482_MOESM4_ESM.docx]

|  | N= | Absolute error (mmol/24h; mean ± SD) |
| --- | --- | --- |
| Blood pump CVVH (ml/min) |  |  |
| ≤160 | 150 | 3.58 ± 4.19 |
| 161-180 | 73 | 5.41 ± 5.90 |
| 181-200 | 63 | 4.65 ± 4.30 |
| 201-220 | 126 | 5.80 ± 4.72 |
| >220 | 26 | 4.71 ± 8.00 |
| p-value |  | 0.01 |
| Plasma calcium (ionized) (mmol/l) |  |  |
| ≤ 0.9 | 7 | 5.63 ± 3.97 |
| 0.9-1.1 | 58 | 4.94 ± 5.93 |
| 1.1-1.3 | 324 | 4.83 ± 4.85 |
| >1.3 | 49 | 4.45 ± 3.24 |
| p-value |  | 0.9 |
| Percentage predilution (%) |  |  |
| ≤60 | 74 | 4.58 ± 4.84 |
| 60-70 | 291 | 4.80 ± 4.67 |
| 70-80 | 73 | 5.07 ± 5.47 |
| p-value |  | 0.8 |
| Citrate dose (mmol/L.blood) |  |  |
| 2.0-2.4 | 284 | 4.71 ± 4.36 |
| 2.41-2.8 | 99 | 5.07 ± 5.94 |
| 3.0 | 54 | 4.82 ± 5.03 |
| p-value |  | 0.8 |
| CVVH filter |  |  |
| HF1400 |  | 3.99 ± 4.25 |
| ST150 | 44 | 5.58 ± 5.22 |
| p-value |  | 0.01 |

Supplementary table 3. Performance of our newly developed model in the validation set in subgroups based on blood flow CVVH (ml/min), plasma concentration of ionized calcium (mmol/l), percentage predilution, citraat dose (mmol/l in blood in circuit) and used filter. Error is calculated calcium loss minus actual measured calcium loss. Values as mean ± SD. P-values by Oneway ANOVA.
